# Adaptive Potential of Maritime Pine (*Pinus pinaster*) Populations to the Emerging Pitch Canker Pathogen, *Fusarium circinatum*

**DOI:** 10.1371/journal.pone.0114971

**Published:** 2014-12-11

**Authors:** Margarita Elvira-Recuenco, Eugenia Iturritxa, Juan Majada, Ricardo Alia, Rosa Raposo

**Affiliations:** 1 Silviculture and Forest Management Department, Forest Research Center (CIFOR), Instituto Nacional de Investigación y Tecnología Agraria y Alimentaria (INIA), Madrid, Spain; 2 Instituto Vasco de Investigación y Desarrollo Agrario (NEIKER), Granja Modelo-Arkaute, Vitoria-Gasteiz, Spain; 3 Forest and Wood Technology Research Center (CETEMAS), Finca Experimental La Mata, Principado de Asturias, Spain; 4 Forest Ecology and Genetics Department, Forest Research Center (CIFOR), Instituto Nacional de Investigación y Tecnología Agraria y Alimentaria (INIA), Madrid, Spain; 5 Sustainable Forest Management Research Institute, Palencia, Spain; Umeå Plant Science Centre, Umeå University, Sweden

## Abstract

There is a concern on how emerging pests and diseases will affect the distribution range and adaptability of their host species, especially due to different conditions derived from climate change and growing globalization. *Fusarium circinatum,* which causes pitch canker disease in *Pinus* species, is an exotic pathogen of recent introduction in Spain that threatens its maritime pine (*P. pinaster*) stands. To predict the impact this disease will have on the species, we examine host resistance traits and their genetic architecture. Resistance phenotyping was done in a clonal provenance/progeny trial, using three-year-old cuttings artificially inoculated with the pathogen and maintained under controlled environmental conditions. A total number of 670 ramets were assessed, distributed in 10 populations, with a total of 47 families, 2 to 5 half-sibs per family, and 3–7 ramets per clone. High genetic variation was found at the three hierarchical levels studied: population, family and clone, being both additive and non-additive effects important. Narrow-sense and broad-sense heritability estimates were relatively high, with respective values of 0.43–0.58 and 0.51–0.8, depending on the resistance traits measured (lesion length, lesion length rate, time to wilting, and survival). These values suggest the species' high capacity of evolutionary response to the *F. circinatum* pathogen. A population originated in Northern Spain was the most resistant, while another from Morocco was the most susceptible. The total number of plants that did not show lesion development or presented a small lesion (length<30 mm) was 224 out of 670, indicating a high proportion of resistant trees in the offspring within the analyzed populations. We found large differences among populations and considerable genetic variation within populations, which should allow, through natural or artificial selection, the successful adaptation of maritime pine to pitch canker disease.

## Introduction

Under global change scenarios, the future of many plant species, and especially their ability to cope, is a subject of great concern. An indirect effect of these scenarios of changing climatic conditions and increasingly globalized trade on plant health is an accelerated introduction of new competitive and invasive pathogens and pests [1]. Many studies have addressed how climate change will affect the adaptability and distribution range of plant species and the potential distribution of invasive species [2], [3], but not many have addressed how emerging pests and diseases will affect their host species' adaptability and range distribution.

It is now generally accepted that the primary mode of introduction of exotic pathogen and insect species is human and product mobility, now increasingly conducted at a global scale [4]. As a consequence, over the last century forest pathogens and insect pests have been increasingly reported [5], [6], [7], [8]. Forest pathogen invasions, in particular, have grown exponentially in Europe in the last four decades, with introductions mainly from North America, but recently also from Asia [9]. Such invasions may affect the dominant tree species in a particular forest ecosystem, reducing its presence and initiating a cascading effect over the ecology [10], function and value of that forest [7], [11]. Notable examples include *Ophiosthoma novo-ulmi* in Western Europe, which had a devastating impact on mature elm trees (*Ulmus minor*) in the 1970s [12]; *Chalara fraxinea*, which has caused extensive ash (*Fraxinus excelsior*) dieback throughout Europe since the 1990s [13]; and *Phytophthora ramorum*, a generalist pathogen of recent introduction in Europe and America [14], [15].

In Mediterranean ecosystems, the exotic forest pathogens affecting trees species are numerous [16]. One of them is *Fusarium circinatum* Nirenberg and O′Donnel, which causes pitch canker disease in pine species. This pathogen, probably a Mexican native species [17], [18], first became important in Southeastern USA on slash and loblolly pines (*Pinus elliottii* and *P. taeda*, respectively), and then extended to Monterey pine (*P. radiata* D. Don) in California in 1986 [18], first exclusively in plantations, but currently also in native stands [19]. Nowadays pitch canker disease is considered one of the most important pine diseases worldwide [20].

The pathogen was first detected in Europe in *P. radiata* and *P. pinaster* nurseries in Northern Spain [21]. Later, a survey on the same area confirmed its presence in *P. radiata* plantations and in a single young (two-year-old) *P. pinaster* plantation [22]. Afterwards, the disease has been reported in France (in a garden on *Pinus* sp.) [23], Italy (in urban parks on *P. halepensis* and *P. pinea*) [24], and Portugal (in nurseries on *P. radiata* and *P. pinaster*) [25]. At present, the disease is restricted in Spain to the Atlantic area [26], where most Monterey plantations are grown. However, in a recent survey conducted in the Basque Country area (northern Spain) to evaluate the disease's incidence on pine species other than *P. radiata*, the presence of *F. circinatum* was confirmed in one *P. pinaster* adult plantation [27], although not on any other pine species. This record has important implications regarding the pathosystem affected because, unlike *P. radiata, P. pinaster* is a Mediterranean native species. In this new context, increasing our knowledge of the impact the pathogen has on the future distribution and adaptive potential of maritime pine populations becomes of utmost importance.

Maritime pine (*Pinus pinaster* Ait) is a Mediterranean forest tree species of high economic and ecological importance, distributed in Western Europe and northern Africa, where it grows in a wide range of habitats with contrasting gene pools. The symptoms of a *F. circinatum* infection are branch dieback and, as the disease progresses, the apparition of stem cankers with exudation of abundant resin. Multiple branch infections may occur, accompanied by severe canopy defoliation. The disease causes a decrease in tree growth, reproduction success and survival rates [20]. Crucially, it may well represent a new selective factor in the evolution of maritime pine populations if there is a heritable variation for resistance [28]. In fact, there is evidence supporting the existence of sufficient genetic variation, and a high degree of genetic control of resistance to *F. circinatum* to allow selection to operate in most pine species: *P. radiata* presents a heritability in the range of 0.34–0.78 depending on the population of origin [29], and *P. taeda* one of 0.27 [30]. There is also evidence of genetic variation in resistance to *F. circinatum* in other Mediterranean pine species (*P. pinea*, *P. halepensis*, *P. nigra*, *P. uncinata* [31], and *P. canariensis* [32]), based on differential responses when seedlings are artificially inoculated with the pathogen. *P pinaster* families from Northwestern Spain present heritabilities in the range of 0.18–0.45 [33]. Up to now, only two pine species (*P. tecunumanii* and *P. maximinoi*) showed very little family variation, being highly resistant to pitch canker disease, with a narrow-sense heritability of less than 0.06 [34].

Disease resistance traits are defined as host traits that reduce the extent, growth or multiplication of pathogen infection [35], and their genetic variation in natural populations is usually estimated by quantitative variation in visual symptoms [36]. Accordingly, disease resistance is only properly estimated when disease symptoms are a consequence of pathogen growth [36]. Similarly, plant tolerance to pathogen infection is defined as the host's ability to reduce the effect of infection on its fitness [35]. Growth and colonization by *F. circinatum* was recently described in artificially inoculated pine seedlings [37]. Three phases were established in plants, correlated to symptom expression, differentiating pathogen growth (measured by relative fungal to pine DNA): (1) exponential phase, visually correspondent with absence of external symptoms; (2) transition phase, during which necrosis from inoculation point was visible; and (3) stationary phase, in which stabilization of the fungal biomass occurred and wilting became apparent. At this time, a generalized collapse of the traqueids and their surrounding living cells occurred at the inoculation point, producing dead foliage above that point.

We explore the evolutionary and adaptability potential to an exotic pathogen (causal agent of pitch canker disease) of a native Mediterranean pine (*Pinus pinaster*) with a strong population structure (populations differentiated throughout its native range, where quantitative traits showed adaptive differentiation and adaptation to different selective factors) [38], [39], [40]. To achieve this goal, we estimate the genetic variation for resistance to *F. circinatum* in a clonal provenance/progeny trial under controlled environmental conditions, to study population genetic variation along a latitudinal cline at three hierarchical levels: population, family and individual. These quantitative experiments separate genetic from environmental variation, which is the basis to estimate genetic variation among and within populations, and to dissect the genetic architecture of a resistance trait. Pitch canker resistance was evaluated in three-year-old inoculated seedlings by lesion length, lesion length rate –a measure of pathogen growth rate-, and time to wilting –a measure of the time taken by the pathogen to reach the stationary phase. We also describe a previous experiment in which clones within populations were phenotyped for disease resistance in order to make a first assessment of genetic variation in *P. pinaster*.

## Materials and Methods

### Ethics statement

*P. pinaster* is not a protected or endangered species and therefore, specific permissions were not required for collecting seed lots. Locations for clonal populations derived from the seed lots are specified in Table 1 and S1 Table.

**Table 1:** Location, climatic data and altitude of the *Pinus pinaster* populations tested in 2012 (except CDVO) and 2013 trials.

Code	Population	Location	A	LA	LO	AMT	MTWM	MTCM	AP
ARMY	Armayán-Asturias	N Spain	498	43.304802	−6.458273	11.8	24.0	2.0	1112
ASPE	Arenas de San Pedro-Avila	Central Spain	733	40.194822	−5.116213	14.2	33.4	1.2	1318
CDVO	Cadavedo-Asturias	N Spain	210	43.539965	−6.417847	13.2	22.0	5.0	1316
COCA	Coca-Segovia	Central Spain	800	41.254705	−4.497827	12.3	31.2	−0.6	454
MIMI	Mimizan-Landes	SW France	37	44.134167	−1.303167	13.3	24.8	3.2	1235
ORIA	Oria-Almería	SE Spain	1223	37.531165	−2.351138	13.1	30.7	0.4	357
PLEU	Pleucadeuc-Morbihan	W France	80	47.781194	−2.343667	11.2	21.9	2.5	804
PTOV	Puerto de Vega-Asturias	N Spain	121	43.547949	−6.631375	13.4	22.6	4.9	1283
SCRI	San Cipriano-Pontevedra	NW Spain	300	42.118331	−8.364440	12.3	26.0	2.7	1600
TAMR	Tamrabta-Middle Atlas	Morocco	1758	33.600000	−5.016667	10.7	30.4	−4.6	745

A: Altitude (m), LA: Latitude (°), LO: Longitude (°), AMT: Annual mean temperature (°C), MTWM: maximum temperature of the warmest month, MTCM: minimum temperature of the coldest month, AP: annual precipitation (mm).

### Plant and fungal material

Plant material for inoculation tests originated from two clonal collections produced and maintained in SERIDA, Asturias (Spain) as follows: twenty-four autochthonous populations of *P. pinaster* from Spain, France, Italy, Portugal and Morocco (Table 1 and S1 Table) were selected along a latitudinal cline, and cones were collected from 10–30 mother trees. A first clonal collection structured in population and clones within population (no family structure) was derived by using one seedling per mother tree cone from each of the mother trees. These seedlings were used for clonal propagation following the protocol previously described [41], [42]. Five clonal replicates per individual seedling were used.

A second clonal collection structured in population, families (in a number of 5) within populations, and individuals (in number of 5) within families (see [43]) was established. Five seeds per family (mother tree cone) were sown, and the resulting half-siblings from each of the five families from each of the 10 populations were used for clonal propagation of 7 clonal replicates per individual half-sibling.

Inoculation tests with *F. circinatum* were performed in level 2 biosafety greenhouses, using three-year old plants, allowing them to acclimatize for two months prior to the test. Selection of suitable plants for inoculation with *F. circinatum* was done discarding those with weak growth and appearance. The *F. circinatum* isolate used for inoculation was CECT20759 (Spanish Culture Collection, Valencia), representative of the fungal population analyzed in the Basque country [44], isolated from *P. radiata* and identified as Mat-1 mating type.

A first inoculation test was performed in 2012 (Vitoria-Gasteiz, Northern Spain), including 365 ramets (73 clones, 5 replicates per clone) from 23 populations (Table 1 except CDVO population and S1 Table) from the first clonal collection. A second inoculation test was performed in 2013 (Madrid, Central Spain), using 678 ramets (165 clones, with 3–7 replicates per clone), derived from 47 families (2–5 half-sibs per family), from 10 populations (4–5 families per population, except PLEU -3 families-) from the second clonal collection (Table 1). Twenty-two clones from nine of these populations were evaluated both in the 2012 and 2013 experiments.

### Experimental design and plant inoculation

Plants were distributed in a randomized design in 2012 and in a row-column (14 rows and 50 columns) randomized design in 2013. Greenhouse conditions in the 2012 trial (from 29 April to 17 June) were set at 18°C±5, and 55–60% relative humidity, while conditions in the 2013 trial (from 27 March to 10 May) were 22°C±5, and 45–65% relative humidity. Neither received supplemental lighting.

For both trials, the *F. circinatum* isolate was grown on potato dextrose agar (PDA, Oxoid) for 7 days at 22°C in darkness. Inoculation point per plant was marked in the stem around the middle point of the second year growth. The inoculum was scrapped off the agar with a sterile pin and stabbed into the stem parallel to the stem axis. Plants were put into plastic bags previously made wet during 48 h in order to achieve high humidity. Fifteen *P. radiata* sapling plants were used as a positive control, inoculated with *F. circinatum* and their disease progression followed along the experiment. Seven plants in the 2012 trial and 10 plants in the 2013 trial coming from the progeny material were used as control to be mock-inoculated (no pathogen).

### Assessment of variables related to *F. circinatum* resistance and plant growth

In the 2013 trial, plants with symptoms of wilting (loss of turgor in shoot tip above inoculation point) were recorded when first seen at 9, 17, 23, 29, 35, 38, 43 or 49 days post inoculation (dpi). When wilting symptoms resulted in plant dieback (death of shoot above inoculation point), the plant was collected. At 49 dpi, the remaining plants without symptoms or with dieback were harvested. Plants with wilting symptoms were left additional days until dieback occurred and then were harvested. After harvesting, lesion length, plant height and diameter were measured, aerial biomass was weighted and plants oven-dried at 60°C until constant weight to measure root and stem dry weight. Plant resistance to *F. circinatum* was evaluated by: (1) Lesion Length (LL) measured with a caliper after removing bark surrounding inoculation point (in mm) and recorded at sampling date; (2) Lesion length growth per day (LLRATE) calculated as the ratio of LL to time of dieback; (3) Time (days) to symptoms of wilting above inoculation point (T_W); and (4) Survival (SV), percentage of plants per population not showing wilting at 49dpi. Plant growth was evaluated by: (5) Aerial Fresh Weight (AFW) (in g), including stem, branches and needles; (6) Stem Diameter at inoculation point (D) (in mm); (7) Height (H) (in mm); (8) Root Dry Weight (RDW) (in g); and (9) Stem Dry Weight (SDW) (in g).

In the 2012 trial, plants were harvested only at the end of experiment, at 44 dpi, and lesion length (LL) was recorded. Presence of wilting was recorded at 23 and 44 dpi.

Stem pieces around inoculation point of plants with lesions (with or without wilting) were randomly selected for reisolation of *F. circinatum*. All asymptomatic plants (i.e. without lesions or wilting) were sampled (1 plant in 2012 and 8 plants in 2013 trials). Reisolation was done as described by Iturritxa et al. [31]

### Statistical analysis

#### Survival analysis (2013 trial)

Seedlings showing no symptoms of wilting at the end of the experiment were considered right-censored observations. To compare the survival curves among populations, Kaplan-Meier estimates of the survivor function were computed, and a log-rank test was applied. Analysis was done using LIFETEST procedure in SAS 9.3 [45].

#### Quantitative genetic analysis

For the 2012 trial, a mixed model was used to analyze the LL variable:
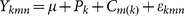
Where Y_kmn_ is the value of the variable for the n^th^ ramet (clonal replicate) from the m^th^ clone (individual) within the p^th^ population; μ, the overall mean of the variable; P_k,_ the effect of the k^th^ population; C_m(K)_, the effect of the m^th^ clone within the k^th^ population and *ε*_kmn,_ the residual.

For the 2013 trial, and for all the variables, the following mixed model was used:
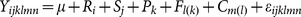
Where Y_ijklmn_ is the value of the variable for the n^th^ ramet (clonal replicate) from the m^th^ clone (individual) within the l^th^ family within the p^th^ population in the i^th^ row and the j^th^ column; μ, the overall mean of the variable; R_i_, the effect of the i^th^ row, S_j_ the effect of the j^th^ column; P_k,_ the effect of the k^th^ population; F_l(k)_, the effect of the l^th^ family within the k^th^ population; C_m(l)_, the effect of the m^th^ clone within the l^ty^ family within the k^th^ population; and *ε*_ijklmn_, the residual.

Variables considered as fixed effects were Population (in both trials), Row and Column (in the 2013 trial). Family (in the 2013 trial) and Clone (in both trials) were considered random effects. Residuals were assumed independent and normally distributed (0, V_e_) for all the variables except SV, which was analyzed using a binomial logit link function (see [46]). In the 2012 trial, the variable LL was log_10_ transformed for the statistical analysis. In the 2013 trial, variables LL and LLRATE were squared root transformed for the statistical analysis. Variance components and significance of effects, best linear unbiased estimator (BLUE) values for populations, and best linear unbiased predictors (BLUP) for families and clones were calculated by restricted maximum likelihood, using the REML algorithm implemented in the ASREML program [46]. This program analyzes the significance of random effects by Likelihood Ratio Test.

Differences among predicted population means were compared by Tukey-Kramer test of multiple comparison means. For the binary variable SV, mean comparison for each population pair was done using Wilcoxon method. All tests were performed with a significance level of 0.05.

Population effect was extracted to estimate the genetic parameters within population so that all families and clones were considered as belonging to the same population.

Clonal repeatability was computed for the 2012 experiment as:
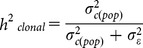
Where 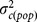 is the clone variance within population, 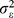 is the residual variance,

Narrow sense (h^2^) and broad sense (H^2^) heritability were computed in the 2013 experiment for each trait as follows:
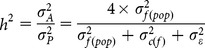


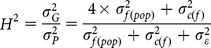
where 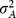 is the additive variance, estimated by 
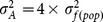
 assuming that seedlings from the same family were half-sibs, 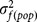 is the family variance within population, 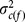 is the clone (individual) variance within family, 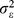 is the residual variance, 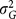 is the genetic variance, and 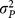 is the phenotypic variance. Heritability estimates for survival (variable analyzed by binomial logit) were also done using these formulas and therefore, estimates were obtained in a binomial scale. Conversion to the liability scale will not be presented since heritability estimates were higher than 1.

Standard errors of heritability were computed using a Taylor series approximation [46].

We estimated the stability of disease response across experiments using the subset of 22 clones that had been phenotyped in the two experiments (2012 and 2013). Two measures of stability were used: correlation coefficient between Best Linear Unbiased Predictor (BLUP) of lesion length untransformed value for clones at the two sites, and Kendall's coefficient of rank correlation [47].

#### Correlation between traits

Correlations between traits measured in the 2013 trial were estimated by calculating Pearson's correlation coefficients on population Best Linear Unbiased Estimator (BLUE) values (population correlations), on family Best Linear Unbiased Predictor (BLUP) values (additive genetic correlations), and on family BLUP plus clone BLUP values (total genetic correlations).

## Results

### Disease progression over time

Reisolation of *F. circinatum* from the lesion was achieved in all plants selected for it. *F. circinatum* was not reisolated for those plants not showing any lesion at inoculation point or aerial symptoms (1 plant in the 2012 trial and 8 plants in the 2013 trial), and inoculation was assumed to have failed. These plants were excluded from subsequent statistical and genetic analyses.

In the 2012 trial, percentage of plants presenting wilting was 54% (197 plants out of 364) at 44 dpi. At 23 dpi, 27% of plants presented symptoms of wilting above inoculation point or had already developed dieback. Lesion length measured at end of experiment (i.e. at 44 dpi) for all plants in the trial was >30 mm for a 32.6% of plants.

**Figure 1:**
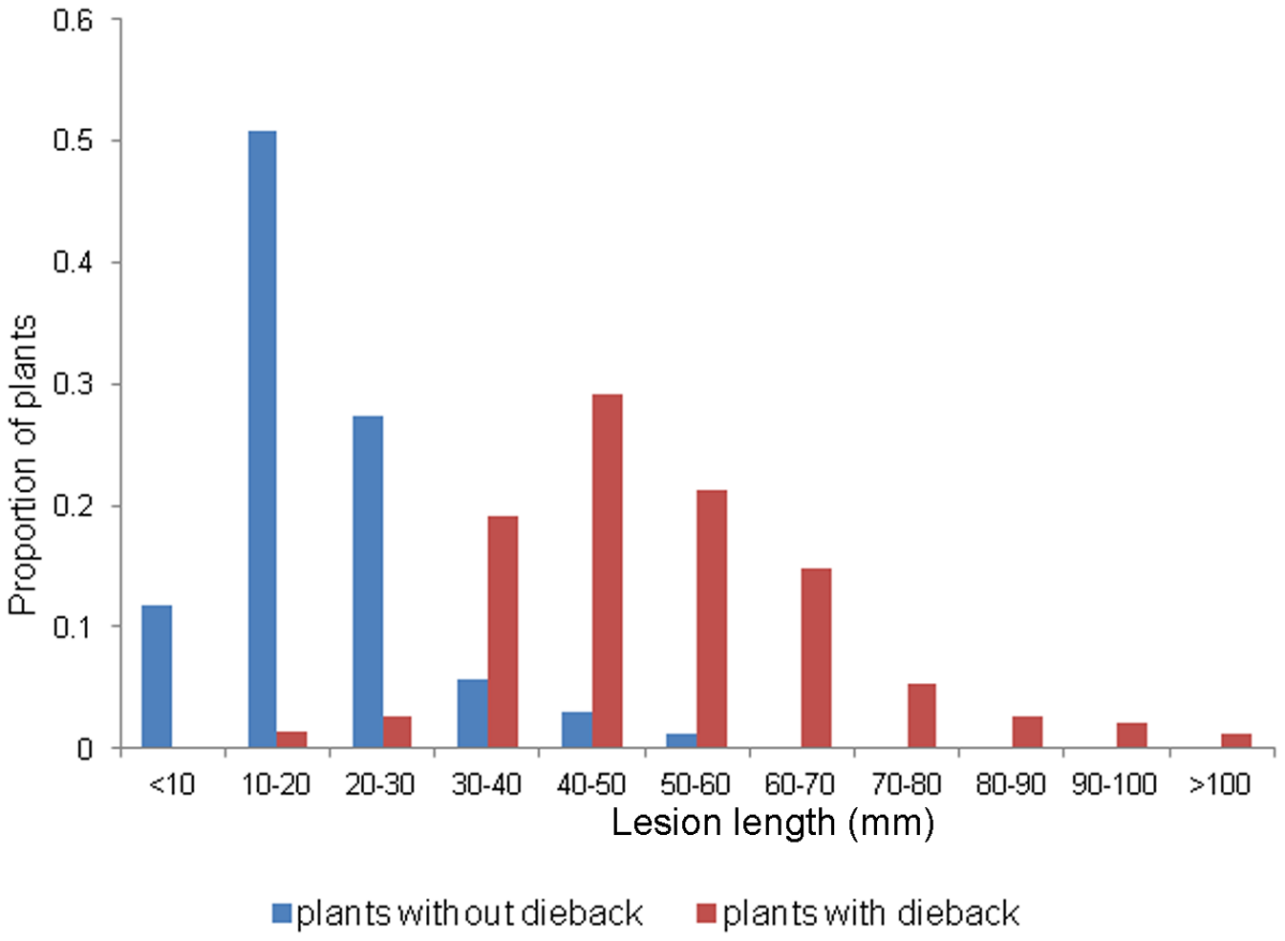
Proportion of plants of *Pinus pinaster* inoculated with *Fusarium circinatum* that did (in red) or did not show (in blue) dieback for intervals of lesion length measured at the sampling date (2013 trial).

In the 2013 trial, total percentage of plants presenting wilting along the experiment was 67% (452 plants out of 670), resulting in dieback in all cases. First symptom of disease observed was wilting above inoculation point, becoming dieback in a period of 3 to12 days. Plants (7% of total number) began showing symptoms of wilting at 17 dpi, and then 0.7% of total plants developed dieback at 20 dpi, the moment of collection. Peak of number of plants presenting wilting symptoms was recorded at 23 dpi (23.1% of total plants), and peak of dieback at 29 dpi. Lesion length was>30 mm for 34% of total number of plants. At 49 dpi, 218 plants had survived and showed no symptoms of wilting. However, lesion length was over 30 mm for about 10% of plants (Fig. 1). Plants with dieback and presenting lesions over 30 mm were 96%, with no plants presenting lesions under 10 mm. According to these results, we established that plants with lesion lengths under 30 mm were resistant, and those over 30mm, susceptible. Mean lesion length for plants with dieback ranged from 50–70 mm when collected at 23 dpi, to 45–56 mm at 35 dpi, and 38–57 mm at 49 dpi, that is, plants that died earlier showed longer lesion lengths. Mean lesion length for plants without lesions by population ranged from 13 to 21 mm at the end of the experiment.

### Population variation

In the 2012 trial, the predicted mean of lesion length was log_10_LL = 1.328±0.333, with a population significant effect (F value = 1.65, with df_num_  = 22 and df_den_  = 120; p-value = 0.045). The number of populations tested was 23, higher than in the 2013 trial, being TAMR the second most susceptible population. In the 2013 trial, the highest LL and LLRATE values and the lowest T_W and SV corresponded to TAMR, i.e. the most susceptible population, being PTOV the most resistant among the studied populations (Table 2). Lesion length predicted mean was √LL = 6.075±0.580, and population effect was significant for all variables related to *F. circinatum* resistance (LL, LLRATE, T_W, and SV) (Table 3). Differences in wilting among populations became obvious after maximum number of wilted plants was reached at 23 dpi. Ratio of wilted plants decreased differently among provenances until 35 dpi. From this date to the end of the experiment at 49 dpi this ratio increased for some populations (COCA, ASPE and PLEU) while decreasing for others (ARMY, CDVO, MIMI and TAMR), and was stable for the rest of them (ORIA, SCRI and PTOV).

**Table 2:** Means of predicted values according to the statistical mixed model, standard errors (in brackets) and statistical significance (with letters) for each trait at the population level (2013 trial).

Trait	ARMY	ASPE	CDVO	COCA	MIMI	ORIA	PLEU	PTOV	SCRI	TAMR
**LL**	35.459 (4.150)	34.675 (5.025)	37.037 (3.930)	39.787 (6.001)	42.662 (5.092)	44.600 (4.117)	40.119 (6.775)	33.032 (4.371)	34.933 (4.296)	56.012 (4.464)
	Bc	bc	Bc	bc	bc	b	bc	c	bc	a
**LLRATE**	1.12 (0.158)	1.07 (0.192)	1.06 (0.149)	1.16 (0.229)	1.28 (0.194)	1.23 (0.157)	1.13 (0.259)	0.88 (0.167)	1.03 (0.164)	1.82 (0.170)
	Cd	cd	cd	bc	b	bc	cd	d	cd	a
**T_W**	36.179 (2.187)	36.380 (2.658)	37.453 (2.046)	34.924 (3.227)	33.896 (2.695)	33.520 (2.149)	34.796 (3.656)	41.365 (2.314)	38.469 (2.267)	25.945 (2.349)
	B	bc	bc	ab	b	b	bc	c	bc	a
**SV**	0.410 (0.125)	0.322 (0.125)	0.419 (0.120)	0.208 (0.108)	0.204 (0.091)	0.190 (0.073)	0.29 (0.148)	0.618 (0.142)	0.504 (0.142)	0.009 (0.006)
	Bc	bc	bc	ab	ab	ab	abc	c	bc	a
**D**	3.795 (0.171)	3.464 (0.215)	3.724 (0.159)	3.362 (0.261)	3.459 (0.214)	3.905 (0.168)	3.584 (0.295)	3.7977 (0.182)	3.954 (0.178)	3.876 (0.182)
	A	a	a	a	a	a	a	a	a	a
**H**	22.037 (1.285)	22.841 (1.521)	22.801 (1.199)	19.402 (1.843)	23.102 (1.543)	22.037 (1.250)	25.770 (2.114)	25.499 (1.354)	26.470 (1.328)	20.654 (1.387)
	A	abc	a	a	ab	a	cd	cd	de	a
**AFW**	12.293 (1.821)	10.242 (2.136)	12.266 (1.731)	9.659 (2.580)	12.007 (2.211)	11.438 (1.794)	14.739 (2.926)	14.995 (1.909)	15.214 (1.881)	9.860 (1.964)
	A	a	a	a	a	a	a	a	a	a
**RDW**	1.876 (0.160)	1.085 (0.190)	1.915 (0.151)	1.435 (0.235)	2.006 (0.195)	1.603 (0.158)	1.899 (0.267)	2.004 (0.168)	2.257 (0.168)	1.571 (0.172)
	Bc	a	cd	ab	bcd	b	bcd	cd	d	ab
**SDW**	1.996 (0.251)	1.688 (0.293)	2.156 (0.240)	1.725 (0.353)	2.239 (0.301)	2.184 (0.250)	2.422 (0.408)	2.413 (0.258)	2.639 (0.261)	2.261 (0.268)
	A	a	a	a	a	a	a	a	a	a

LL: lesion length (mm) of plants with or without dieback at sampling date, LLRATE: lesion length growth per day, T**_**W: time (days) to first symptoms of wilting above inoculation point, SV: proportion of plants without wilting at the end of experiment, D: stem diameter (mm) at the inoculation point, H: stem height (mm), AFW: aerial fresh weight (g), RDW: root dry weight (g), SDW: stem dry weight (g). Means with the same letter did not differ significantly, according to Tukey-Kramer test (p<0.05) for all variables except SV, in this case Wilcoxon test was used. Populations described in Table 1.

**Table 3:** Variance components, narrow-sense and broad-sense heritability with their standard error for each trait obtained from the mixed model in the 10 studied populations of *P. pinaster* (2013 trial).

Trait	F_PR_	P_PR_	Vf_am_	SE_fam_	Pf_am_	V_C_	SE_C_	P_C_	V_R_	SE_R_	h^2^ ± SE	H^2^ ± SE
LL	2.42	0.029	0.317	0.166	0.028	0.486	0.152	0.0007	2.038	0.138	0.447±0.216	0.618±0.197
LLRATE	2.48	0.026	0.014	0.008	0.037	0.026	0.007	<.0001	0.090	0.006	0.430±0.224	0.630±0.201
T_W	3.00	0.009	9.680	7.048	0.085	25.647	7.295	<.0001	91.465	6.158	-	0.508±0.189
SV	2.88	0.011	0.750	0.461	0.052	1.115	0.416	0.004	3.289	-	*0.582±0.321*	*0.800±0.282*
D	0.99	0.465	0.05	0.043	0.121	0.143	0.048	0.001	0.757	0.050	-	0.361±0.158
H	2.20	0.045	2.753	2.30	0.115	14.556	2.752	<.0001	15.71	1.066	-	0.774±0.220
AFW	0.98	0.473	8.458	4.603	0.033	15.632	4.001	<.0001	47.276	3.169	0.474±0.237	0.693±0.212
RDW	3.39	0.004	0.054	0.036	0.065	0.169	0.037	<.0001	0.30	0.021	-	0.739±0.222
SDW	1.04	0.429	0.181	0.085	0.017	0.279	0.065	<.0001	0.556	0.041	0.714±0.293	0.988±0.257

Traits as described in Table 2. F_PR_: F distribution value to test population factor (fixed effect in the mixed model) for each trait, with df_num_ = 9, df_den_ = 36, P_PR_: p-value of population significance, V_fam_: variance between families, SE_fam_: standard error of V_fam_, P_fam_: p-value of V_fam_ significance, V_c_: variance between clones, SE_C_: standard error of V_C_, P_C_: p-value of Vc significance, V_R_: residual variance, SE_R_: standard error of V_R,_ h^2^: narrow sense heritability, H^2^: broad sense heritability, SE: standard error of heritability. All tests were performed with significance level of 0.05. The variable SV was analyzed by a binomial logit and heritabilities are indicated in the observed binomial scale (in cursive).

**Figure 2:**
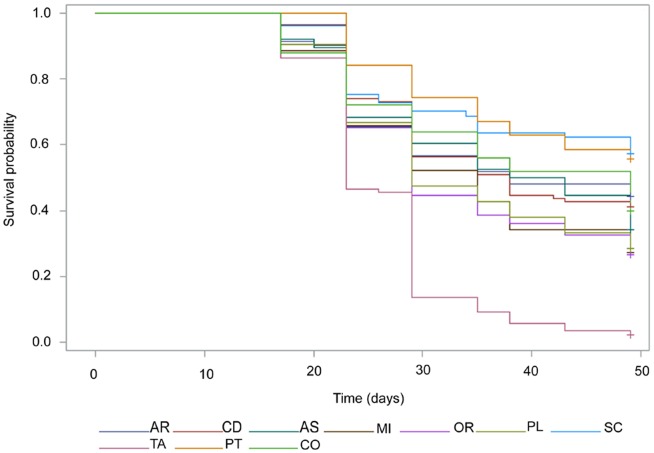
Survival function for the event of wilting by populations of *P. pinaster* plants inoculated with *F. circinatum* at time 0 and estimated by the Kaplan-Meier method (2013 trial).

The cumulative proportion of plants showing symptoms of wilting was analyzed using survival analysis based on Kaplan-Meir estimates. They were significantly different among populations (p<0.0001) according to the log-rank test for homogeneity of survival curves (Fig. 2). Plants from TAMR population showed significantly more wilting, followed by ORIA population. PTOV and SCRI were the ones that survived pitch canker disease more, their plants with wilting not reaching 50% at the end of the experiment at 49 dpi. At 23 dpi, 50% of plants had already wilted in TAMR population, becoming 75% at 29 dpi. All populations reached 25% of plants presenting wilting at 23 dpi except SCRI (26 dpi) and PTOV (29 dpi). With the exception of the most susceptible and the resistant populations, they were not ranked in the same way as when using the T_W variable. It should be noted that in survival analysis plants that remain alive at the end of the experiment are censored, while in T_W the value is assumed to be the final date of the experiment (49 dpi).

When plants were grouped by presence/absence of dieback (i.e. dead/alive), there were no significant differences among populations for all the variables analyzed within each group of plants (data not shown).

For the traits related to growth characters, only RDW and H were different among populations (p<0.05) (Table 2). SCRI presented the highest values for all growth traits measured. It is interesting to notice that mean values for TAMR and PTOV, the most susceptible and resistant populations, were not significantly different from the extreme values.

### Within-population genetic variation

Genetic variation in pitch canker resistance was observed among clones (genotypes) within populations (V_clonal_  = 0.060, SE = 0.019, p-value  = 0.0007) in the 2012 trial. Clonal repeatability was 0.301±0.073, indicating that genetic effects accounted for nearly 30% of phenotypic variation.

In the 2013 trial, where plants were structured in families and clones, an additive variance component was estimated to calculate narrow-sense heritability (h^2^) (Table 3). Significant levels of additive genetic variation were observed for the LL and LLRATE traits, with narrow-sense heritability values around 0.45. Estimate for SV was 0.58, higher than for the LL and LLRATE traits (Table 3). Their standard errors were high, due to the relatively small number of families analyzed. Non-additive genetic variation (among individuals) within families was highly significant for *F. circinatum* resistance traits, and broad-sense heritability values (H^2^) were 0.51-0.80.

High levels of variation were observed among families within populations. For the most resistant populations, PTOV and SCRI, the range value (difference in survival between the lowest and highest value among families) was 80%, while in the most susceptible population, TAMR, there was almost no variation in survival among families with a maximum range value of 6%.

### Correlations between traits

In the 2013 trial, total genetic correlations between growth and pitch canker resistance traits were significant for all variables except plant height (H), which was significantly correlated only with lesion length (LL) (Table 4). Highly significant additive and total genetic correlations were observed among the variables related to *F. circinatum* resistance (Table 4). Additive genetic correlations had values above 0.70, and were negative both between LL and SV, and between LL and T_W. Correlations at population level followed the same pattern with values above 0.90. All growth traits were negatively correlated with LL and LLRATE and positively correlated with T_W and SV, indicating that plants growing better in terms of diameter and biomass, showed shorter lesion lengths and survived more proportionally and in time. Correlations at population level were significant in SV and AFW. It is interesting to notice that correlation for those traits has the same sign at population level as at additive and total genetic correlations.

**Table 4:** Pearson correlation coefficients among resistance and growth traits for 10 populations, 47 families and 165 clones of *P. pinaster* (2013 trial).

Trait	LL	LLRATE	T_W	SV	D	H	AFW	RDW	SDW
**LL**	1.000	**0.95**	**−0.700**	**−0.848**	**−0.344**	0.252	**−0.376**	−0.150	−0.208
		**0.942**	**−0.677**	**−0.785**	**−0.315**	**0.203**	**−0.375**	**−0.184**	**−0.172**
**LLRATE**	**0.960**	1.000	**−0.814**	**−0.866**	**−0.446**	0.168	**−0.471**	−0.263	**−0.345**
			**−0.80**	**−0.788**	**−0.377**	0.128	**−0.448**	**−0.265**	**−0.269**
**T_W**	**−0.956**	**−0.978**	1.000	**0.896**	**0.589**	0.129	**0.688**	**0.485**	**0.544**
				**0.860**	**0.531**	0.111	**0.637**	**0.446**	**0.450**
**SV**	**−0.964**	**−0.98**	**0.976**	1.000	**0.514**	0.049	**0.601**	**0.424**	**0.423**
					**0.541**	0.097	**0.612**	**0.416**	**0.413**
**D**	0.151	0.129	−0.027	−0.051	1.000	**0.467**	**0.908**	**0.645**	**0.827**
						**0.418**	**0.867**	**0.610**	**0.786**
**H**	−0.502	−0.542	0.585	0.587	0.311	1.000	**0.590**	**0.537**	**0.702**
							**0.527**	**0.383**	**0.673**
**AFW**	−0.509	−0.573	0.627	**0.634**	0.393	**0.933**	1.000	**0.751**	**0.900**
								**0.708**	**0.880**
**RDW**	−0.221	−0.27	0.365	0.385	0.456	**0.643**	**0.809**	1.000	**0.777**
									**0.713**
**SDW**	0.063	0.02	0.121	0.105	**0.638**	**0.741**	**0.800**	**0.836**	1.000

Lower triangle correlations between population BLUE values. Upper triangle: for each trait, first row correlations between family BLUP values (additive genetic correlation), second row correlations between family plus clone BLUP values (total genetic correlation). Significant correlations (p<0.05) in bold letter. Trait abbreviations in Table 2.

### Genetic stability across trials

Both correlation coefficients indicated there was interaction between clones and experiments performed at two different environmental conditions for LL. Correlation among BLUP estimates from both trials was 0.244 (p-value  = 0.275), and the rank correlation coefficient was 0.439 (p-value  = 0.149), without agreement between clone ranking in both trials. However, although this GE interaction existed for LL, 11 clones were defined as resistant and 5 as susceptible, in both years based on the lesion length. Overall, lesion length was larger in 2013 (mean ± standard error  = 39.41 mm ±3.186 in 2013, and 30.09±2.970 mm in 2012; p-value <0.0001)

## Discussion

The present study revealed strong genetic variation in pitch canker phenotypic disease response at the three levels of variation analyzed: population, family and clone, demonstrating the importance of additive as well as non-additive effects for the traits related to resistance response measured in *P. pinaster*. Other studies have analyzed some of these levels in populations of *Pinus* species [48], in families within a population [49], [50] or at both levels [29]. Moreover, most studies on *F.circinatum-Pinus pinaster* interaction have been limited to seedlings coming from families of selected genotypes from one population [33]. Thus, the results from the present study represent the first report of genetic variation at all three levels in a novel host-pathogen interaction in a *Pinus* species. We provide information that allows to understand the evolutionary forces shaping resistance to the disease, and the future implications of the incidence of this pathogen in a novel host.

Variation in disease resistance to emerging pathogens has been observed in other hosts in natural populations. Clones of European ash (*Fraxinus excelsior*) showed genetic resistance to the emerging pathogen *Chalara fraxinea* [51], and an American chesnut (*Castanea dentata*) to *Cryphonectria parasitica* [52]. Unlike maritime pine populations, native trees are highly susceptible to emerging pathogens, and their effects on them devastating. Species of *Pinus* vary widely in susceptibility to the pathogen *F. circinatum* [27], [31], [34], [53]. *P. radiata* is one of the most susceptible species, while *P. canariensis* and *P. pinea* are among the most resistant, showing no mortality caused by pitch canker disease [32]. In the case of *P. pinaster*, when evaluated on inoculated seedlings, the species showed significantly shorter lesion lengths in comparison with *P. radiata* [31]. This record, together with the overall mortality measured in our study (67%), define maritime pine as moderately susceptible to *F. circinatum*. Maritime pine has also shown genetic variation in response to other pathogens, such as *Diplodia pinea* [27]; and to *Melampsora pinitorqua*, a rust pathogen [54].

We found differences in pathogen response at the population level. This study showed that PTOV population, from Northern Spain (Table 1) was the most resistant, while TAMR, from Morocco, was the most susceptible to pitch canker disease. Therefore, we expect that in some locations (especially TAMR origin) the impact of the disease will be high and the proportion of resistant trees small. Maritime pine is a species that exhibits high differentiation among populations in growth and drought related traits (e.g. isotopic discrimination, cavitation resistance) [39], [40], [43], [55], or even historic and demographic events [56] that might be also related to pitch canker resistance. We explored the possibility of a relation between genetic variation for disease resistance and growth traits, but the absence of correlation at the population level (Table 4) ruled out this hypothesis. Also, the genetic stability we found across experiments suggests the existence of some structural properties in the genotypes of the resistance mechanisms. However, the coincident ranking of population for drought related traits [40], [43], [55] –being TAMR the population with the lowest survival rate, and SCRI and PTOV among the most resistant-, suggests that resistance is a by-product of adaptation to other evolutionary factors related to drought tolerance. We further explored the correlation between tolerance to drought and resistance to pitch canker disease at the population level to support our hypothesis. Our populations had been previously evaluated by Gaspar et al. for drought tolerance [43], with results for survival at day 100^th^ (S100). The correlation coefficients between: (1) S100 [43] and % of survival to pitch canker disease (SV, this study) was 0.86±0.18 (p-value  = 0.0014); and (2) S100 and time to showing disease symptoms (T_W, this study) was 0.89±0.05 (p-value  = 0.006), indicating a close relationship. In fact, the interaction between drought and disease caused by fungal pathogens is well known. Drought is considered a predisposing factor in tree diseases, i.e. trees under stress are more susceptible to disease [57], [58]. In the case of *F. circinatum*, it has been suggested that this pathogen may play a role in the collapse of the xylem [37], [59]. Accordingly, infection and drought might share an underlying mechanism related to the plant's capacity to withstand water stress. Consequences of this emerging disease could be more dramatic in *P. pinaster*'s southern range of distribution, not only because TAMR population is the most susceptible to the disease, but also because it is the most vulnerable to climatic change [60]. The potential distribution of pitch canker disease is, however, expected to extend to northern Europe because of increasing average minimum temperatures [61], a limiting factor of the pathogen's distribution according to the CLIMEX model [62].

Plants that grew best in terms of diameter and biomass showed shorter lesion lengths and survived more in proportion and with time, as their significant genetic correlations between growth and pitch canker resistance traits (Table 4). In other words, there were positive relationships between growth and resistance-related traits. These results are not consistent with current theories that point out that defenses against disease reduce plant resources for growth and reproduction [63]. The possibility that plant biomass measured at the end of our experiment may have been affected by the infection process (i.e. plants not under severe disease would grow faster), would point at a positive relationship. However, our experiment was not designed to measure said relationship, so further studies would be required in order to do so. Many conifers species experiment an induced increase in resin flow as an effective defense mechanism against insects and pathogens [64]. This is the case of maritime pine′ response to *Hylobius abietis* (pine weevil) attacks, when high resin content followed plant infestation [65]. Monterey pine plants responded to *F. circinatum* infection by increasing both the number of resin ducts and the amount of resin flow [37], [66], although the function of the induced resin is not obvious, and there is evidence not only that resin production is not protective against the pathogen [67], but that the fungus is able to grow inside resin ducts [37] and stimulate resin production [37], [67].

Interestingly, we found 10% of plants evaluated developing lesions longer than 30 mm (which made them pitch canker susceptible) (Fig. 1), but not showing any symptoms of wilting or dieback (and were therefore considered disease tolerant) when assuming that the plants would not die if the trial were extended in time. The occurrence of naturally infected Monterey pine (*P. radiata*) plants that do not show symptoms has been described somewhere else [19], and it represents the extreme of total tolerance to pitch canker disease. Disease tolerance can reduce the effect of pathogen selection on plant evolution [36], since plants can support stronger infections without fitness reduction. Therefore, the pathogen tolerance detected in *P. pinaster* populations may have significant consequences on their evolutionary responses to pitch canker disease.

Survival is the trait with the highest values for heritability, which could indicate its being a good parameter in the evaluation and selection of plants for resistance to pitch canker disease, but the resulting proportion of tolerant plants may give misleading results. Traits of resistance and tolerance to pathogens may very well be indicative of different levels of genetic control. Plant resistance traits are those that reduce pathogen growth while tolerance traits are those that reduce the effect of infection on plant fitness. According to this definition, the characters evaluated in our study (i.e. lesion length and time before visible symptoms) are directly related to pathogen growth and thus, are both traits of resistance. Survival, in contrast, is a trait that evaluates disease tolerance or both tolerance and resistance.

Narrow-sense (h^2^) and broad-sense (H^2^) heritability estimates were high, indicating a high capacity of evolutionary or breeding response of the species to the *F. circinatum* pathogen. Values for h^2^ and H^2^ were, respectively, 0.43–0.58 and 0.51–0.8, depending on the resistance traits measured (lesion length rate, time to wilting and survival). Other authors found a similar narrow-sense heritability for tree mortality caused by *F. circinatum* in Atlantic populations of *P. pinaster* (h^2^ = 0.45) [33], similar to the one observed for different *P. radiata* populations (values of h^2^ = 0.34–0.78 [29], [53], and higher than for *P. taeda* (h^2^ = 0.27 and H^2^ = 0.43)[30]. According to the present study, there is enough variation for the different populations to evolve. This study showed that, regardless of population origin, at least 50% of the individuals belonging to 14 families survived out of the 47 tested, and the total number of plants that did not show lesion development or that showed small lesions (length<30 mm) was 224 of 670. Therefore, we expect that trees from these populations will produce resistant offspring to pitch canker disease with very low to non-existent signs of disease. Previous studies showed a high correlation between lesion length measured in the artificially inoculated seedlings and frequency of infections in the field [32]. The phenotypic disease resistance response varied with the environmental trial conditions but, even so, resistant (LL<30 mm) and susceptible (LL>30 mm) clones were similarly distinguished.

The potential for adaptive evolution of quantitative traits depends on the amplitude of their additive genetic variance [68]. Under the hypothetical scenario of pitch canker disease spreading over the Mediterranean region, we expect that natural selection will favor resistant trees, given the relatively high level of additive variation and the high narrow-sense heritability. In the short term, the extent of the disease's damage will be limited by the resistance shown by individual trees [69], and there will be some locations (especially TAMR origin) where the impact will be higher. But even in these populations selection will be expected to favor surviving trees. Furthermore, according to Gordon et al. [70], in areas where pitch canker was well established, Monterey pine trees tended to be more resistant than trees in areas where the disease was of more recent occurrence. Their findings support that SIR (systemic acquired resistance) occurs in *P. radiata* and is contributing to a moderation in the impact of pitch canker disease under natural conditions. Natural selection may have different intensities in different populations or gene flow may be restricted among host populations, both cases leading to significant spatial genetic structure. Some studies provided evidence for a direct host response caused by the pathogen, in which the response may vary with pathogen distribution, pathogen virulence and environmental parameters affecting disease risk [71]. For example, Hamilton et al. [72] showed that spatial variation in disease risk is a driving force for adaptive differentiation across the geographic distribution of *Eucaliptus globulus* in relation to Mycosphaerella leaf disease. A recent disease risk model developed for pitch canker disease occurrence in Northern Spain [73] revealed that summer precipitation is positively correlated with the disease's occurrence, and is the most relevant parameter in the model. That is, moisture may be effectively limiting the distribution of pitch canker disease to areas with higher precipitation during summer [73], and may be causing a reduced disease incidence or severity, becoming a possible driving factor in adaptive evolution. However, some questions need to be addressed: changes over time in the level of resistance and the relations between the environmental variables on the rate of disease spread. These two main factors need more experimental research to predict more precisely the likely incidence of pitch canker disease in maritime pine over different areas.

In summary, our results showed that *P. pinaster* is a species with moderate to high genetic variation for resistance to the pitch canker pathogen, with important additive effects that lead us to expect evolutionary responses to the disease at the three levels studied. We also presented some evidence that resistance to pitch canker disease could be related to other biotic and abiotic stresses.

## Supporting Information

S1 Table **Location, climatic data and altitude of the** ***Pinus pinaster*** **populations tested in the 2012 trial along with populations of** **Table 1** **.**
(DOCX)
