# Supplementary material for: Adaptive Potential of Maritime Pine (Pinus pinaster) Populations to the Emerging Pitch Canker Pathogen, Fusarium circinatum
Source: PLoS One. 2014 Dec 11;9(12):e114971. doi: 10.1371/journal.pone.0114971 (PMC4263721; doi:10.1371/journal.pone.0114971)
Supplement: S1 Table — Location, climatic data and altitude of the Pinus pinaster populations tested in the 2012 trial along with populations of Table 1 . (DOCX) [file pone.0114971.s001.docx]

Table S1. Location, climatic data and altitude of the *Pinus pinaster* populations tested in the 2012 trial along with populations of Table 1

| Population | Location | A | LA | LO | AMT | MTWM | MTCM | AP |
| --- | --- | --- | --- | --- | --- | --- | --- | --- |
| Hourtin-Landes | SW France | 26 | 45.18333333 | -1.15 | 12.8 | 24.7 | 2.8 | 980 |
| Leverdon-Landes | SW France | 11 | 45.55194444 | -1.09055556 | 12.8 | 24.8 | 2.9 | 893 |
| Olonne sur Mer-Vendée | W France | 13 | 46.56591667 | -1.83061111 | 12.2 | 23.2 | 3.2 | 845 |
| Petrocq-Landes | SW France | 31 | 44.06397222 | -1.29955556 | 13.3 | 24.9 | 3.1 | 1248 |
| Sierra de Barcia-Asturias | N Spain | 240 | 43.52802139 | -6.49321942 | 13.0 | 22.4 | 4.7 | 1339 |
| Leiria | W Portugal | 20 | 39.78333333 | -8.9575 | 15.4 | 24.4 | 7.4 | 811 |
| Pineta | Central Corsica | 750 | 41.97269167 | 9.038366667 | 15.5 | 26.5 | 6.7 | 583 |
| Pinia | E Corsica | 10 | 42.02108333 | 9.464861111 | 15.6 | 26.9 | 6.3 | 580 |
| Valdemaqueda-Madrid | Central Spain | 890 | 40.51625208 | -4.31113172 | 12.1 | 29.2 | 1.2 | 681 |
| Bayubas de Abajo-Soria | Central Spain | 998 | 41.52297286 | -2.87743056 | 10.6 | 29.6 | -1.4 | 553 |
| San Leonardo-Soria | Central Spain | 1096 | 41.83458153 | -3.06272011 | 9.3 | 27.9 | -2.7 | 753 |
| Quatretonda-Valencia | E Spain | 435 | 38.97164506 | -0.35884372 | 15.3 | 30.3 | 3.8 | 777 |
| La Bisbal-Girona | NE Spain | 224 | 41.91416667 | 3.050555556 | 15.0 | 26.7 | 4.7 | 679 |
| Sidi-Meskour-High Arlas | Morocco | 2050 | 31.506175 | -6.99453333 | 12.3 | 33.2 | -4.3 | 486 |
|  |  |  |  |  |  |  |  |  |

A: Altitude (m), LA: Latitude (°), LO: Longitude (°), AMT: Annual mean temperature (ºC), MTWM: maximum temperature of the warmest month, MTCM: minimum temperature of the coldest month, AP: annual precipitation (mm)
